# Gender Difference in the Association between Environmental Tobacco Smoke and Birth Weight in Africa

**DOI:** 10.3390/ijerph15071409

**Published:** 2018-07-04

**Authors:** Patrick Opiyo Owili, Miriam Adoyo Muga, Hsien-Wen Kuo

**Affiliations:** 1 Department of Public Health, School of Health Sciences, University of Eastern Africa, Baraton, Eldoret 30100, Kenya; owilip@ueab.ac.ke; 2 International Ph.D. Program in Environmental Science and Technology, Institute of Environmental and Occupational Health Sciences, School of Medicine, National Yang-Ming University, Taipei 112, Taiwan; 3 Graduate Programs, School of Health Sciences, University of Eastern Africa, Baraton, Eldoret 30100, Kenya; mugam@ueab.ac.ke; 4 Department of Foods, Nutrition and Dietetics, School of Science and Technology, University of Eastern Africa, Baraton, Eldoret 30100, Kenya; 5 Institute of Environmental and Occupational Health Sciences, School of Medicine, National Yang-Ming University, Taipei 112, Taiwan

**Keywords:** environmental tobacco smoke, low birthweight, gender, Sub-Saharan Africa

## Abstract

The adverse health effects of exposure to environmental tobacco smoke (ETS) on children are well-documented, and yet, gender difference in low birthweight among newborns whose mothers were exposed to ETS during pregnancy still remains contentious. We therefore explored the association between ETS exposure and risk of low birthweight, and further determined the gender difference in the association between exposure to ETS during pregnancy and birth weight in Africa. The Demographic Health Surveys of 23 African countries with information on 208,027 newborns were used. The associations between exposure to ETS and birth weight was estimated using multiple logistic regression models. Exposure to ETS increased the risk of low birthweight in Africa (adjusted odds ratio (OR) = 1.06; 95% Confidence Interval (CI): 1.02–1.10). A stratified analysis, by gender, revealed that male newborns whose mothers were exposed to ETS were 1.08 (95% CI: 1.02–1.14) times more likely to be low in birthweight than those whose mothers were not exposed, with those exposed weekly (adjusted OR = 1.17; 95% CI: 1.01–1.35) and daily (adjusted OR = 1.06; 95% CI: 1.01–1.12) being more likely to have low birthweight. Exposure to ETS is significantly associated with low birthweight in Africa, mainly among male newborns. Gender could possibly be a modifier, and hence, research on biological plausibility is necessary. Moreover, a public health promotion on behavioral changes is likely to have a positive impact on newborns’ health.

## 1. Introduction

Even though active and passive exposure to environmental tobacco smoke (ETS) during pregnancy has been associated with a wide-range of health risks, including still birth, prematurity, and miscarriage [1,2,3], many women in Sub-Saharan Africa (SSA) are still exposed to cigarette smoke in the household during pregnancy. Globally, over 33% of the population is frequently exposed to cigarette smoke actively or passively, and, of all female non-smokers, about 35% were exposed to secondhand tobacco smoke (STS) [4]. In SSA as recently as 2015, however, adult daily smoking prevalence varied greatly between countries, with some countries estimated to be as low as 3% (i.e., Ethiopia) with others as high as 26% (i.e., Sierra Leone) [5]; it was also estimated that about 10% of female non-smokers are exposed to STS in a region where most countries have not implemented 100% smoke-free public health regulations, and exposure to STS is still common [4,5,6]. In 2004, 600,000 deaths were attributed to exposure to STS globally, of which 47% and 28% were women and children, respectively [6]. The adverse health effects of tobacco smoke on children are well-documented [7,8], and include intrauterine or fetal growth restriction, and a considerable decrease in birth weight by over 10 g per cigarette/day [9,10,11,12,13].

A few studies have indicated that there could be gender difference in low birthweight and mortality, with some indicating that males are disadvantaged [14,15,16,17]. Several studies have also analyzed gender as a confounder without considering that it could be a modifier [18,19,20]. Yet, gender difference in low birthweight among newborns whose mothers were exposed to ETS during pregnancy still remains contentious. A study in Japan found that the newborns of smoking mothers were at risk of mean birth weight reduction, and that the risk of restricted fetal growth was only high among male newborns [21]. On the other hand, a study in Germany found the contrary, citing the adverse effect of ETS during pregnancy on the mean birth weight and risk of small-for-gestational-age was higher among females than among males [22]. No study has directly explored this gender difference among children exposed to ETS in Africa, though one did explore the relationship between smoking during pregnancy and low birthweight in South Africa, finding that mothers who were smoking during pregnancy were 2 times more likely to give birth to newborns who were low in weight [23].

The research limitation in Africa is due, in part, to focus on other important health concerns, such as infectious diseases and access to quality delivery care, which is reasonable. However, with the shift of tobacco markets from high- to low-income countries with non-stringent control measures [24], a tobacco epidemic may soon occur in low-income countries, which may result in unprecedented levels of death and illness, thus making researching gender difference and ETS much more urgent. Therefore, our study aims to explore the association between ETS exposure and risk of low birthweight, and to further address the knowledge gap about gender difference and the association between exposure to ETS during pregnancy and birth weight in Africa.

## 2. Materials and Methods

### 2.1. Study Population

Data from a nationally representative household surveys of 23 African countries collected by the Demographic Health Survey (DHS, 2010 to 2014), in collaboration with the Ministry of Health of each country, were used. The cross-sectional interview targeted information about women aged 15–49 years and their babies. A stratified sampling technique, using a two-stage cluster technique, was employed to sample households. Specific details about the sample, data collection, and processing is available in the final report of each country [25]. Eligible mothers were those who had given birth within five years preceding the survey. Excluded from the current analysis were mothers who gave birth, but, due to incomplete data reporting about their babies, were ineligible for the study. The total number of children in the study were 208,027, represented as follows: Benin (2011/12; *n* = 11,423), Bukina Faso (2010; *n* = 14,292), Burundi (2010; *n* = 7213), Comoros (2012; *n* = 2537), Congo (2011/12; *n* = 7789), Côte d’Ivoire (Ivory Coast, 2011/12; *n* = 6426), Democratic Republic of Congo (DRC, 2013/14; *n* = 17,255), Ethiopia (2011; *n* = 11,048), Gabon (2012; *n* = 4044), Gambia (2013; *n* = 7218), Guinea (2012; *n* = 6474), Kenya (2014; *n* = 8916), Liberia (2013; *n* = 5474), Mali (2012/13; *n* = 8391), Mozambique (2011; *n* = 9589), Nigeria (2013; *n* = 29,529), Namibia (2013; *n* = 2096), Rwanda (2010; *n* = 8291), Sierra Leone (2013; *n* = 10,171), Togo (2013/14; *n* = 6522), Uganda (2011; *n* = 7508), Zambia (2013/14; *n* = 11,049), and Zimbabwe (2010/11; *n* = 4772).

### 2.2. Measurements

Two questionnaires were administered to participating mothers. The first questionnaire was used to collect household information, and the second was used to collect information about the mother and baby. *Outcome variable*: low birthweight was defined as babies born weighing less than 2500 grams, as confirmed in the baby’s health card. For the purpose of our analyses, we dichotomized birth weight (i.e., 1 = low birthweight; 0 = otherwise). *Exposure variable*: A question about frequency of exposure to ETS in the household had five responses: “never”, “daily”, “weekly”, “monthly”, and “less than monthly.” In our analysis, exposure to ETS responses were combined, and the indicator was dichotomized (i.e., 1 = exposed to ETS; 0 = otherwise). *Covariates*: We controlled for the child’s characteristics (i.e., gender and birth order), and the mother’s characteristics, which included mother’s age, education (i.e., “no education”, “primary”, and “secondary and higher”), and mother’s occupation (i.e., “unemployed”, “professional/office-related”, “sales”, “farming”, and “other services”). Other covariates included country, residence (i.e., “urban” or “rural”), each country’s provinces, family size, household wealth index (i.e., “poorest”, “poor”, “middle”, “rich”, and “richest”), father’s occupation (i.e., “unemployed”, “professional/office-related”, “sales”, “farming”, and “other services”), cooking fuel (i.e., “clean” or “pollutant”), the proxy measure of the level of exposure to household air pollution, and kitchen location (i.e., “in house”, “separate building”, and “outdoor”). Pollutant cooking fuel included “biomass”, “charcoal”, “coal/lignite”, and “kerosene” while clean fuel included “gas” and “electricity.”

### 2.3. Statistical Analysis

The association between exposure to ETS in the household and birth weight was estimated using multiple logistic regression models. The crude and adjusted odds ratios (ORs) and 95% confidence intervals (Cis) for low birthweight were generated. We developed three models in our analyses. The first model was unadjusted, while the second was adjusted for the child’s and mother’s characteristics (i.e., child’s gender, birth order, mother’s age, mother’s education, and mother’s occupation). The third model was adjusted for all the indicators (i.e., model 2 plus country, residence, family size, wealth index, father’s occupation, cooking fuel, and kitchen location). A stratified analysis by gender was then performed to estimate the effect of exposure to ETS in the household on the weight of the newborn. In all our analyses, we employed a complex survey analysis technique to adjust for the sampling design, which incorporated the primary sampling unit, cluster, country, and sampling weights. Chi-Square was also used to test for the group difference of categorical variables while generalized linear regression was employed for continuous variables. All statistical analyses were performed using the Stata software package version 13.1 [26].

### 2.4. Ethics Approval and Consent to Participate

Ethics committees of each country approved the study before conducting the survey, and the participants were required to sign an informed consent before participating. All the data were anonymized, and ethical standards of the DHS were followed [27]. Additional ethical approval was also obtained from the Institutional Review Board (IRB), approval number YM106020E, of National Yang-Ming University in Taipei, Taiwan.

## 3. Results

Table 1 presents the characteristics of the participants. Twenty-two percent of the mothers in our study were exposed to ETS in the household, with 22.7% of those who had low birthweight being exposed ETS. The majority of mothers exposed to ETS were exposed daily (18.0%). Mothers from Sierra Leone (44.9%) who lived in rural areas (23.8%) and were involved in farming (27.8%) and used pollutant cooking fuel (22.2%) were exposed to ETS. All the indicators were statistically significant at *p* < 0.05, except for gender, which was not different in exposure to ETS.

Table 2 shows the crude and adjusted results of the associations between ETS and low birthweight in Africa. Newborns of mothers who were exposed to ETS in the household were 5% more likely to have low birthweight than those who were not exposed before adjustment. After adjustment of the child’s and the mother’s characteristics the association was not significant (model 2); but, with the adjustment of all the indicators (model 3), ETS exposure significantly increased the risk of low birthweight in Africa (adjusted OR = 1.06; 95% CI: 1.02–1.10). The risk of low birthweight was also significant among those who were exposed daily to ETS (adjusted OR = 1.05; 95% CI: 1.01–1.10). Also, in comparing male and female newborns, an increase in the likelihood of low birthweight was observed among female newborns than male newborns (adjusted OR = 1.26; 95% CI: 1.22–1.30). Newborns who were also born in houses with indoor kitchens were 1.07 (95% CI: 1.01–1.13) times more likely to have low birthweight. The socioeconomic indicators also showed that mothers who were highly educated were less likely to have low birthweight (adjusted OR = 0.78; 95% CI: 0.70–0.86) than those without education. The richest participants were also less likely to have low birthweight (adjusted OR = 0.73; 95% CI: 0.67–0.79) than the poorest.

When we stratified the newborns by gender, a significant positive association between maternal ETS exposure and the risk of low birthweight was found only among male newborns (Table 3). Male newborns whose mothers were exposed to ETS were 1.08 (95% CI: 1.02–1.14) times more likely to be low in birthweight than those whose mothers were not exposed, with those who were weekly (adjusted OR = 1.17; 95% CI: 1.01–1.35) and daily (adjusted OR = 1.06; 95% CI: 1.01–1.12) exposed to ETS being significantly more likely to have low birthweight. However, the relationship among female newborns was not statistically significant. Nevertheless, mothers who were older and were educated were less likely to have newborns of both genders with low birthweight.

## 4. Discussion

We determined the association between exposure to ETS and low birthweight in Africa and subsequently explored gender differences and the likelihood of low birthweight, finding that exposure to ETS in the household during pregnancy increases the risk of low birth weight, especially among male newborns, who were 8% more likely to have low birthweight if their mothers were exposed to ETS in the household when compared to babies whose mothers were not. Frequency of exposure also contributed greatly to the risk of low birthweight. Our findings are consistent with a study conducted in Japan, which found a significant inverse relationship between maternal exposure to ETS and birth weight only among male newborns [21], but differed with the study conducted in Germany, which found that the negative effect of ETS on birth weight was greater among females than among males [22].

As smoking prevalence is increasing in some countries in Africa due to a shift of tobacco markets from high- to low-income countries [5,24], adverse health outcomes such as low birthweight may be inevitable. Our study found that mothers in Africa exposed to ETS were 1.06 times more likely to have newborns with low birthweight. Our study results support the findings of other studies in different populations that have also shown exposure to ETS was positively associated with the risk of low birthweight [28,29,30,31,32]. Countries with high exposure rate to ETS, such as Sierra Leone (44.9%), should consider reevaluating and intensifying tobacco control campaigns as laid down in the World Health Organization Framework Convention on Tobacco Control [33].

Regarding the gender differences in the association between ETS exposure and risk of low birthweight in Africa, our findings are consistent with other authors, who also found that male newborns were the most at risk of low birthweight [15,16,17,21]. However, because other authors found a contrary opinion [22], with no clear explanation for the gender difference, it is therefore necessary to develop a biologically plausible study to explore the reasons for gender difference. Nevertheless, some authors have alluded to the interference of tobacco toxins with trophoblast cell activity and the biological functions of cells regulating enzyme activity and protein metabolism being the main reason of reduced fetal growth and weight [34]. Others have however suspected chemical pollutants interfering with the action of testosterone, an anabolic steroid which is a vital male hormone that helps fetuses to gain weight in the process of growth, and more especially, severely affecting male fetuses than female [35]. Other possible reasons could be high rates of newborns who are small for gestational age in low- and middle-income countries (i.e., estimated at 32.4 million), of whom, some who were born at term had low birthweight already (i.e., estimated at 10.6 million) [36]. However, the possible reasons for high male susceptibility still remain unclear in the literature and these inconsistencies among studies may also be explained, at least in part, by the differences in other factors such as environment, lifestyle, sociodemographic, and genetics of the study populations. For instance, our study found that mothers who had formal education and were older were less likely to have newborns of low birthweight, regardless of the gender difference, unlike the birth order.

With the continued evidence about the health effects of ETS, policymakers in Africa should integrate tobacco control measures with maternal, newborn, and child health care programs. Cigarette smokers should be encouraged to avoid smoking indoors, and campaign programs on smoking cessations should also be enhanced.

Implementing stringent tobacco control laws and creating smoke-free environments is also important in limiting exposure to ETS, as is encouraging mothers to take precautionary measures to prevent exposure to ETS during pregnancy.

## 5. Limitations and Strengths

Our study had several limitations, which included an inadequate tobacco exposure assessment that was only obtained using a questionnaire without measurement of duration of maternal exposure and cotinine levels in the household; consequently, we may have underestimated the true effect of ETS in Africa. Secondly, our findings should be interpreted with caution because we used a cross-sectional dataset, and operated under the assumption that ETS over time was even over time, and so causality could not be ascertained. A prospective cohort study would be appropriate with accurate measurement of exposure to ETS toxins. Lastly, we were unable to adjust for several unmeasured confounders, such as biological factors, that could explain our finding, because data was collected for general national policy purposes, and is therefore limited. Nevertheless, our study adds to the body of knowledge about the health effect of ETS on gender difference. It is the first study of its kind, with a wide range of representative populations in Africa, unlike other studies that are country-specific [21,22], thus increases the generalizability of our findings to the African population.

## 6. Conclusions

Exposure to ETS in the household is significantly associated with low birthweight in Africa, particularly among male newborns. Our results find that gender could possibly be a modifier rather than a confounder. More research to explore the biological plausibility of our findings is appropriate. Public health promotions disseminating general behavioral recommendations is likely to have a positive impact on maternal, newborn, and child health.

## 7. Availability of Data and Materials

The data that support the findings of this study are available from Demographic Health Survey but restrictions apply to the availability of these data, which were used under license for the current study, and so are not publicly available. Data are however available from the authors upon reasonable request and with permission of Demographic Health Survey.

## Figures and Tables

**Table 1:** Characteristics of participants by exposure to environmental tobacco smoke in Africa (Demographic Health Survey (DHS), 2010–2014).

		Total *n*	Not Exposed *n* (%_wt_)	Exposed to ETS *n* (%_wt_)	*p*-Value ^a^
All		208,027	160,722 (78.0)	47,305 (22.0) ^b^	
Birth weight					
	≥Normal	175,019	135,603 (78.2)	39,416 (21.8)	0.022
	Low birthweight	33,008	25,119 (77.3)	7889 (22.7)	
Gender					
	Male	105,451	81,621 (78.2)	23,830 (21.8)	0.157
	Female	102,576	79,101 (77.9)	23,475 (22.1)	
Birth order, ***M* (SD)**		3.8 (2.4)	3.8 (2.4)	4.0 (2.4)	<0.001
Mother’s age					
	≤ 19	9585	7314 (77.6)	2271 (22.5)	<0.001
	20–29	101,505	78,980 (78.7)	22,525 (21.3)	
	30–39	77,188	59,467 (77.7)	17,721 (22.3)	
	≥40	19,749	14,961 (76.2)	4788 (23.8)	
Mother’s occupation					
	Not employed	62,561	49,294 (80.1)	13,267 (19.9)	<0.001
	Professionals	5867	5171 (89.2)	696 (10.8)	
	Sales	39,493	32,197 (82.2)	7296 (17.8)	
	Farming	76,304	54,866 (72.1)	21,438 (27.8)	
	Other services	23,802	19,194 (81.2)	4608 (18.8)	
Country					
	Bukina Faso	14,292	10,260 (71.3)	4032 (28.8)	<0.001
	Benin	11,423	9901 (87.2)	1522 (12.9)	
	Burundi	7213	5065 (69.2)	2148 (30.8)	
	DRC	17,255	11,758 (69.2)	5497 (30.8)	
	Congo	7789	5735 (78.1)	2054 (21.9)	
	Côte d’Ivoire ^c^	6426	4549 (71.1)	1877 (28.9)	
	Ethiopia	11,048	8889 (87.4)	2159 (12.6)	
	Gabon	4044	2729 (74.9)	1315 (25.1)	
	Gambia	7218	4944 (69.2)	2274 (30.8)	
	Guinea	6474	4621 (71.1)	1853 (28.9)	
	Kenya	8916	7700 (86.0)	1216 (14.0)	
	Comoros	2537	1779 (71.5)	758 (28.5)	
	Liberia	5474	4452 (84.4)	1022 (15.6)	
	Mali	8391	6594 (79.4)	1797 (20.6)	
	Mozambique	9589	6948 (71.9)	2641 (28.1)	
	Nigeria	29,529	27,557 (93.2)	1972 (6.8)	
	Namibia	2096	1455 (73.8)	641 (26.2)	
	Rwanda	8291	6700 (80.7)	1591 (19.3)	
	Sierra Leone	10,171	5796 (55.1)	4375 (44.9)	
	Togo	6522	5138 (81.4)	1384 (18.6)	
	Uganda	7508	5690 (75.3)	1818 (24.7)	
	Zambia	11,049	8916 (81.4)	2133 (18.6)	
	Zimbabwe	4772	3546 (73.6)	1226 (26.4)	
Residence					
	Urban	59,109	47,930 (82.4)	11,179 (17.6)	<0.001
	Rural	148,918	112,792 (76.2)	36,126 (23.8)	
Family size, ***M* (SD)**		7.0 (4.2)	6.9 (3.9)	7.5 (4.9)	<0.001
Wealth index					
	Poorest	54,164	39,571 (73.8)	14,593 (26.2)	<0.001
	Poorer	45,907	34,376 (75.0)	11,531 (25.0)	
	Middle	40,807	31,606 (77.7)	9201 (22.3)	
	Richer	36,503	29,390 (81.0)	7113 (19.0)	
	Richest	30,646	25,779 (85.3)	4867 (14.7)	
Father’s occupation					
	Not employed	18,755	14,535 (77.7)	4220 (22.3)	<0.001
	Professionals	18,712	15,980 (85.9)	2732 (14.2)	
	Sales	20,161	16,934 (84.3)	3227 (15.7)	
	Agriculture	96,984	71,091 (74.2)	25,893 (25.8)	
	Other services	53,415	42,182 (79.8)	11,233 (20.2)	
Cooking fuel					
	Clean	8502	6979 (83.3)	1523 (16.7)	<0.001
	Pollutant	199,525	153,743 (77.8)	45,782 (22.2)	
Kitchen location					
	Separate building	77,622	59,180 (76.8)	18,442 (23.2)	<0.001
	In-house	56,930	45,968 (82.1)	10,962 (17.9)	
	Outdoor	73,475	55,574 (75.9)	17,901 (24.1)	

^a^, Chi-Square was used to test for the group difference of categorical variables while generalized linear regression was employed for continuous variables; ^b^, ETS exposure frequency: monthly (*n* = 3178 (1.5%)), weekly (*n* = 5096 (2.5%)) and daily (*n* = 39,031 (18.0%)); ^c^, Also known as Ivory Coast; DRC, Democratic Republic of Congo; ETS, environmental tobacco smoke; *M*, mean; *SD*, standard deviation; *n*, number; %_wt_, weighted percentage.

**Table 2:** Crude and adjusted odds ratios of logistic regression analyses for low birthweight in Africa (DHS, 2010–2014).

		Model 1	Model 2	Model 3
		COR (95% CI)	AOR (95% CI)	AOR (95% CI)
Exposed to ETS (Ref: No)		1.05 (1.01, 1.10) *	1.04 (0.99, 1.08)	1.06 (1.02, 1.10) **
ETS exposure frequency (Ref: No)				
	Monthly	1.10 (0.97, 1.26)	1.12 (0.98, 1.27)	1.07 (0.93, 1.22)
	Weekly	1.07 (0.96, 1.19)	1.08 (0.97, 1.19)	1.09 (0.98, 1.20)
	Daily	1.04 (1.00, 1.09) *	1.03 (0.99, 1.08)	1.05 (1.01, 1.10) *
Gender (Ref: Male)		1.25 (1.22, 1.29) ***	1.25 (1.22, 1.29) ***	1.26 (1.22, 1.30) ***
Birth order **(in 1 unit increments)**		0.99 (0.98, 1.00)	1.00 (0.99, 1.01)	0.99 (0.98, 1.00)
Mother’s age group (≤19 years)				
	20–29	0.80 (0.74, 0.86) ***	0.80 (0.75, 0.86) ***	0.79 (0.74, 0.85) ***
	30–39	0.72 (0.67, 0.77) ***	0.71 (0.66, 0.77) ***	0.72 (0.66, 0.78) ***
	≥40	0.79 (0.73, 0.86) ***	0.76 (0.68, 0.84) ***	0.78 (0.70, 0.86) ***
Mother’s education (Ref: No education)				
	Primary	0.82 (0.79, 0.87) ***	0.82 (0.79, 0.86) ***	0.86 (0.82, 0.91) ***
	Secondary	0.74 (0.71, 0.79) ***	0.73 (0.69, 0.77) ***	0.83 (0.78, 0.89) ***
	Higher	0.64 (0.56, 0.74) ***	0.64 (0.54, 0.74) ***	0.71 (0.60, 0.83) ***
Mother’s occupation (Ref: unemployed)				
	Professional/office-related	0.75 (0.41, 0.50) ***	0.98 (0.86, 1.12)	0.98 (0.85, 1.12)
	Sales	0.86 (0.91, 0.98) ***	0.88 (0.83, 0.93) ***	0.97 (0.92, 1.03)
	Farming	0.90 (1.10, 1.17) ***	0.88 (0.84, 0.93) ***	0.92 (0.88, 0.97) ***
	Other services	0.98 (0.93, 1.02)	1.02 (0.96, 1.09)	1.05 (0.98, 1.12)
Country (Ref: Benin)				
	Bukina Faso	0.96 (0.86, 1.08)		1.01 (0.90, 1.14)
	Burundi	1.32 (1.17, 1.48) ***		1.38 (1.23, 1.56) ***
	DRC	0.89 (0.78, 1.01)		1.01 (0.88, 1.15)
	Congo	0.89 (0.76, 1.05)		0.94 (0.81, 1.10)
	Côte d’Ivoire ^a^	1.14 (0.98, 1.33)		1.24 (1.06, 1.45) **
	Ethiopia	2.61 (2.31, 2.95) ***		2.78 (2.46, 3.14) ***
	Gabon	1.42 (1.19, 1.69) ***		1.55 (1.27, 1.88) ***
	Gambia	1.68 (1.47, 1.93) ***		1.78 (1.55, 2.05) ***
	Guinea	0.96 (0.83, 1.10)		1.03 (0.90, 1.18)
	Kenya	1.12 (0.98, 1.27)		1.28 (1.12, 1.47) ***
	Comoros	1.85 (1.57, 2.19) ***		1.75 (1.47, 2.08) ***
	Liberia	1.60 (1.40, 1.83) ***		1.61 (1.40, 1.84) ***
	Mali	1.00 (0.86, 1.16)		0.88 (0.72, 1.08)
	Mozambique	0.96 (0.85, 1.10)		1.05 (0.93, 1.20)
	Nigeria	1.15 (1.03, 1.28) *		1.15 (1.03, 1.28) *
	Namibia	1.41 (1.18, 1.68) ***		1.60 (1.33, 1.93) ***
	Rwanda	1.14 (1.02, 1.27) *		1.29 (1.15, 1.44) ***
	Sierra Leone	1.29 (1.14, 1.47) ***		1.23 (1.08, 1.40) **
	Togo	1.25 (1.09, 1.42) ***		1.36 (1.19, 1.54) ***
	Uganda	1.72 (1.53, 1.93) ***		1.83 (1.53, 2.19) ***
	Zambia	0.77 (0.69, 0.87) ***		0.88 (0.78, 1.00) *
	Zimbabwe	0.89 (0.77, 1.02)		1.00 (0.86, 1.15)
Residence (Ref: Urban)		1.13 (1.08, 1.19) ***		0.94 (0.88, 1.00) *
Family size **(in 1 unit increments)**		1.00 (0.99, 1.01) ***		1.00 (0.99, 1.01)
Wealth index (Ref: poorest)				
	Poorer	0.87 (0.83, 0.92) ***		0.88 (0.84, 0.93) ***
	Middle	0.83 (0.79, 0.88) ***		0.85 (0.80, 0.90) ***
	Richer	0.78 (0.74, 0.83) ***		0.80 (0.75, 0.85) ***
	Richest	0.71 (0.67, 0.76) ***		0.73 (0.67, 0.79) ***
Father’s occupation (Ref: Not employed)				
	Professional/office-related	0.82 (0.75, 0.90) ***		0.94 (0.81, 1.10)
	Sales	0.86 (0.79, 0.93) ***		0.90 (0.78, 1.04)
	Farming	0.96 (0.90, 1.03)		0.90 (0.79, 1.04)
	Other services	0.84 (0.79, 0.91) ***		0.88 (0.77, 1.01)
Pollutant fuel (Ref: Clean fuel)		1.13 (1.03, 1.24) **		1.04 (0.92, 1.17)
Kitchen location (Ref: Separate)				
	In-house	1.11 (1.06, 1.17) ***		1.07 (1.01, 1.13) *
	Outdoor	0.91 (0.87, 0.95) ***		0.99 (0.94, 1.03)

^a^, Also known as Ivory Coast; DRC, Democratic Republic of Congo; ETS, environmental tobacco smoke; COR, crude odds ratio; AOR, adjusted odds ratio; CI, confidence interval; Model 1 is unadjusted; Model 2 is adjusted for child’s gender, birth order, mother’s age, mother’s education, and mother’s occupation; Model 3 is adjusted for model 2 plus country, residence, family size, wealth index, father’s occupation, cooking fuel, and kitchen location; * *p* ≤ 0.05; ** *p* ≤ 0.01; *** *p* ≤ 0.001.

**Table 3:** Adjusted odds ratios of the logistic regression analyses for low birthweight in Africa, stratified by child’s gender.

		Male	Female
		AOR (95% CI) ^a^	AOR (95% CI) ^a^
Exposed to ETS (Ref: No)		1.08 (1.02, 1.14) **	1.04 (0.98, 1.09)
ETS exposure frequency (Ref: No)			
	Monthly	1.11 (0.93, 1.33)	1.02 (0.86, 1.22)
	Weekly	1.17 (1.01, 1.35) *	1.02 (0.89, 1.17)
	Daily	1.06 (1.01, 1.12) *	1.04 (0.99, 1.11)
Birth order **(in 1 unit increments)**		0.97 (0.96, 0.99) **	1.01 (0.99, 1.02)
Mother’s age group (≤19 years)			
	20–29	0.77 (0.70, 0.85) ***	0.81 (0.74, 0.90) ***
	30–39	0.73 (0.65, 0.82) ***	0.72 (0.64, 0.80) ***
	≥40	0.80 (0.69, 0.93) ***	0.76 (0.66, 0.87) ***
Mother’s education (Ref: No education)			
	Primary	0.77 (0.79, 0.89) ***	0.89 (0.84, 0.95) ***
	Secondary	0.73 (0.71, 0.85) ***	0.88 (0.82, 0.95) **
	Higher	0.80 (0.55, 0.86) **	0.72 (0.59, 0.89) **
Mother’s occupation (Ref: unemployed)			
	Professional/office-related	1.04 (0.86, 1.26)	0.93 (0.78, 1.10)
	Sales	1.00 (0.92, 1.08)	0.95 (0.88, 1.02)
	Farming	0.91 (0.85, 0.97) **	0.93 (0.87, 0.99) *
	Other services	1.07 (0.98, 1.16)	1.04 (0.95, 1.13)

^a^, Adjusted for birth order, mother’s age, mother’s education, and mother’s occupation, country, residence, family size, wealth index, father’s occupation, cooking fuel, and kitchen location; ETS, environmental tobacco smoke; AOR, adjusted odds ratio; CI, confidence interval; * *p* ≤ 0.05; ** *p* ≤ 0.01; *** *p* ≤ 0.001.

## Abbreviations

DHS	Demographic Health Survey
DRC	Democratic Republic of Congo
ETS	Environmental Tobacco Smoke
IRB	Institutional Review Board
SSA	Sub-Saharan Africa
STS	Secondhand Tobacco Smoke
